# Presence of CHD1L Over-Expression Is Associated with Aggressive Tumor Biology and Is a Novel Prognostic Biomarker for Patient Survival in Human Breast Cancer

**DOI:** 10.1371/journal.pone.0098673

**Published:** 2014-08-25

**Authors:** Jiayi Wu, Yu Zong, Xiaochun Fei, Xiaosong Chen, Ou Huang, Jianrong He, Weiguo Chen, Yafen Li, Kunwei Shen, Li Zhu

**Affiliations:** 1 Comprehensive Breast Health Center, Shanghai Ruijin Hospital affiliated to Medical School of Shanghai Jiaotong University, Shanghai, China; 2 Pathology Department, Shanghai Ruijin Hospital affiliated to Medical School of Shanghai Jiaotong University, Shanghai, China; The University of Hong Kong, China

## Abstract

**Background:**

The chromodomain helicase/adenosine triphosphatase DNA binding protein 1–like gene (CHD1L) is a recently identified oncogene localized at 1q21. CHD1L protein over-expression in primary hepatocellular carcinoma is correlated with enhanced apoptosis inhibition, reduced chemosensitivity and shortened patient survival. However, CHD1L protein status or mRNA expression in breast cancer and its clinical significance remain obscure.

**Material and Methods:**

In this study, immunohistochemical staining for CHD1L expression was performed on tissue microarrays containing 179 primary invasive breast cancers and 65 matched normal breast tissue specimens. Clinico-pathological features were collected and compared between different CHD1L statuses. Kaplan-Meier curves were applied to estimate disease-free survival (DFS) and overall survival (OS). Cox regression was used to identify independent prognostic factors. Also, quantitative real-time polymerase chain reaction (QRT-PCR) was employed to evaluate the mRNA level expression of CHD1L in six breast cancer cell lines.

**Results:**

Presence of CHD1L over-expression was observed in 87 of the 179 patients (48.6%), which associated with a younger age (P = 0.011), higher grade (P = 0.004), higher Ki-67 index (P = 0.018) and HER2 over-expression/amplification (P = 0.037). After a median follow-up of 55 months, patients with presence of CHD1L over-expression had significantly poorer DFS (82.6% Vs 76.3%, P = 0.035), but not OS (87.0% Vs 94.9%, P = 0.439). In multivariate analysis, CHD1L status (HR = 2.169, [95%CI, 1.029–4.573], P = 0.042), triple negative subtype (HR = 2.809, [95%CI 1.086–7.264], P = 0.033) and HER2 positive subtype (HR = 5.221, [95%CI 1.788–15.240], P = 0.002) were identified as independent prognostic factors for DFS. In vitro study indicated that relative mRNA expression level of CHD1L was higher in breast cancer cell lines, especially in MDA-MB-231 and LM2-4175, when compared to normal breast epithelial cell line.

**Conclusions:**

Presence of CHD1L over-expression is probably associated with aggressive tumor biology in breast cancer. CHD1L status might be a novel prognostic biomarker for patients with breast cancer.

## Introduction

Breast cancer is the most frequently diagnosed cancer and the leading cause of cancer death in females worldwide, accounting for 23% (1.38 million) of the total new cancer cases and 14% (458,400) of the total cancer deaths in 2008 [1]. Like other solid tumors, the development of breast cancer is associated with the acquisition of genetic and epigenetic alterations and corresponding changes in protein expression that modify normal growth control and survival pathways. In breast cancer, gains in 1q were one of the most frequent genetic alterations and Comparative Genomic Hybridization (CGH) analysis indicated that more than 30% of tumors had regional (>10 Mb) gains in 1q [2]–[4].

The chromodomain helicase/ATPase DNA binding protein 1-like gene (CHD1L), also known as amplified in liver cancer 1 gene (ALC1), was recently identified as a target oncogene within the 1q21 amplicon in HCC [5]. CHD1L belongs to the sucrose nonfermenting 2 (SNF2)-like subfamily of the SNF2 family. SNF2 proteins stabilize or perturb protein–DNA interactions by using the energy released by their DNA-dependent ATPase activity and play important roles in transcriptional regulation, maintenance of chromosome integrity, and DNA repair [6], [7]. CHD1L over-expression occurs in 46% to 86% of HCC and was correlated with venous infiltration, microsatellite tumor nodule formation, advanced tumor stage, poor disease-free survival and poor overall survival [8]–[12]. The ability of CHD1L protein to facilitate carcinogenesis is mainly due to its anti-apoptosis and epithelial-mesenchymal-transition(EMT)-inducing effects [10], [11], which also plays an important role in the development of breast cancer. The status of CHD1L expression in breast cancer and its clinical and prognostic significances is uncertain, but as described above, amplification of 1q has already been frequently detected in primary breast cancer, suggesting that one or more oncogenes within the amplicon may correlated with the development of this breast cancer. Recent gene expression studies have identified breast cancer into at least 5 intrinsic subtypes: Luminal A, Luminal B, HER2-enriched, basal-like, and normal breast-like by gene profiles [13], [14]. Due to the hard access to gene microarrays in clinical practice, a simplified immunohistochemistry (IHC) classification including estrogen receptor (ER), progesterone receptor (PR), human epidermal growth factor receptor-2 (HER2), and Ki-67 index is now considered a surrogate for establishing breast cancer subtypes [15]–[18], which is essential to understand tumor biology, predict prognosis and make treatment decisions. In this study, we examined the protein expression of CHD1L by IHC in a cohort of breast cancer tissues and also identified the correlation of clinicopathological factors, breast cancer subtypes, and prognostic significance. Also, mRNA expression of CHD1L was tested in breast cancer cell lines by quantitative real-time polymerase chain reaction (QRT-PCR) to identify the relationship between expression level and breast cancer subtypes.

## Materials and Methods

### Patients and cell lines

One hundred and seventy-nine primary breast cancer patients treated at Ruijin Hospital, Shanghai Jiaotong University from December 2003 to August 2012 were retrospectively recruited. All patients had early-stage breast cancer with no distant metastasis at diagnosis and were treated with radical surgery (93.9% with mastectomy and 6.1% with breast-conserving surgery). Adjuvant chemotherapy was administered after surgery by the preference of the treating physicians. A total of 139 patients (77.7%) received chemotherapy. Chemotherapy regimens included anthracycline-containing therapies (48.2%, cyclophosphamide plus doxorubicin/epirubicin plus 5-fluorouracil or doxorubicin/epirubicin plus cyclophosphamide), anthracycline and taxane combinations (45.3%, doxorubicin/epirubicin plus cyclophosphamide followed by paclitaxel/docetaxel or docetaxel plus doxorubicin/epirubicin plus cyclophosphamide) or other regimens (6.5%, docetaxel plus cyclophosphamide/carboplatin, cyclophosphamide plus methotrexate plus 5-fluorouracil, etc). Fifty seven percents of the patient underwent radiotherapy and 68.5% were prescribed endocrine therapy, which were also determined according to physician's decision and/or the patient's preference. The proportion of patients treated with adjuvant therapy were similar to the general population in our hospital. Fixed paraffin-embedded tissue samples of all patients were archived for tissue microarray (TMA) construction and IHC test. The following data are also required: age, pathologic tumor size and lymph node status, grade, ER, PR, HER2 and Ki-67 index and follow-up information. All patients were classified into two groups by CHD1L status: CHD1L-over-expressed group and CHD1L-normal group. This retrospective study has been approved by the Ethical Committees of Shanghai Ruijin Hospital. The results of this study do not affect the treatment decision of any patient enrolled. All the clinical and pathological data was collected only after the written informed consent form was obtained from the patient.

Normal breast epithelial cell line MCF-10A and five breast cancer cell lines: BT474, T47D, MDA-MB-468, MDA-MB-231, and MCF-7 were obtained from the American Type Culture Collection (Manassas, VA, USA) and cultured in complete growth medium under recommended conditions. LM2-4175, as described previously by Minn et al. [19], was a kind gift from Dr. Guohong Hu (The Key Laboratory of Stem Cell Biology, Institute of Health Sciences, Shanghai Jiao Tong University School of Medicine), and was cultured in Dulbecco's Modified Eagle Medium (Gibco, Carlsbad, CA, USA) supplemented with 10% fetal bovine serum (Gibco). Among these, BT474, T47D, MDA-MB-468, MDA-MB-231 and MCF-7 enjoy different estrogen receptor status and HER2 gene status, which reflect different subtypes of breast cancer and LM2-4175 represents a metastatic feature.

### Construction of TMA

A total of 179 formalin-fixed and paraffin-embedded breast cancer tissue specimens and 65 matched normal breast tissue specimens were selected from the Surgery Departement of Ruijin Hospital. Matching normal breast tissue was defined as the tissue harvested from the surgical specimen of the same patient and was greater than 5 cm away from the tumor. All tissue samples were sectioned freshly and stained with hematoxylin and eosin. The representative regions of lesion were reviewed carefully and defined by two separate pathologists. Tissue cylinders with a diameter of 0.6 mm were taken from the selected regions of donor blocks and then punched precisely into a recipient paraffin block by tissue arraying instrument. Five-micrometer, consecutive sections of the microarray blocks were made with a microtome.

### Immunohistochemistry (IHC)

IHC staining was performed with the standard streptavidin-biotin-peroxidase complex method. Sections were de-paraffinized and incubated with rabbit polyclonal anti-CHD1L antibody (Sigma, St Louis, MO, USA) in a dilution of 1∶50 at 4°C overnight. CHD1L status was determined by Immunoreactive score (IRS), a semi-quantitative scoring system calculated according to the staining intensity (SI) and percentage of positive cells (PP).SI was defined as 0 = negative,1 = weak, 2 = moderate, and 3 = strong. PP was scored as 0 = <1%; 1 = 1%∼10%; 2 = 11%∼30%; 3 = 31%∼50%; 4 = 51∼80% and 5 = >80% positive cells. With the proportion score times the intensity score, the final score ranged from 0 to 15. Based on previous studies [20], we used the median IRS score as the criterion for presence of CHD1L over-expression.

The ER, PR and HER2 status of surgical specimens were determined by IHC. Positive staining for ER/PR was defined as nuclear staining in ≥1% of tumor cells. HER2 positivity was considered as HER2 3+ by IHC or positive on FISH, whereas cases with 0 to 1+ or 2+ without FISH positivity were regarded as negative. Representative tumor specimens were stained for Ki-67 according to manufacturer's recommendations. The following antibodies were used for the IHC test: ER: clone 1D5 (rabbit monoclonal, Gene), PR: clone PR636 (mouse monoclonal, Dako), HER2: c-erbB-2 (2000∼2008, rabbit polyclonal, Dako) or 4B5 (2009–2012, rabbit monoclonal, Roche), Ki-67: clone MIB-1(mouse monoclonal, Dako). According to St. Gallen Expert Consensus, IHC classification defines Luminal A as ER and/or PR positive, HER2 negative, Ki-67 low (<14%); Luminal B tumors as ER and/or PR positive, HER2 negative,Ki-67 high or as ER and/or PR positive, HER2 over-expressed or amplified, any Ki-67; HER2 positive as HER2 over-expressed or amplified, ER and PR absent; triple negative as ER and PR absent, HER2 negative [15].

### Quantitative real-time polymerase chain reaction (QRT-PCR)

Total RNA was extracted using TRIzol Reagent (Invitrogen, Carlsbad, CA, USA), and reverse transcription was performed using a RevertAid First Strand cDNA Synthesis Kit (Fermentas, Waltham, MA, USA) according to the manufacturer's instructions. QRT-PCR analysis was performed using a SYBR Green PCR Kit (Applied Biosystems, Carlsbad, CA, USA) on an ABI Step One Real-Time PCR Systems (Applied Biosystems). The cycling parameters were 94°C for 30 seconds, 55°C for 30 seconds, and 72°C for 30 seconds for 40 cycles. All QRT-PCR reactions were performed in triplicate. The β-actin gene was used as an endogenous control and MCF-10A as sample reference. Relative mRNA expression of CHD1L was calculated by using standard curve methods.

### Statistical analysis

The Chi-square test was applied to evaluate the relationship between expression of CHD1L and other parameters studied. Fisher's exact test was performed when necessary. DFS interval was defined as the time from the date of the diagnosis of breast cancer to the earliest occurrence of all local, regional, or distant recurrences, all second cancers and contralateral breast cancers, and all deaths. OS was defined as the time from the date of the diagnosis of breast cancer to all deaths whether they were breast cancer–related or not. DFS and OS were estimated using the Kaplan–Meier analysis, and the survival curves were compared using the log-rank test. Multivariate Cox regression analysis with stepwise selection was used to estimate the hazard ratio, 95% confidence interval (CI), and the effects of the clinical and pathological variables. All statistical tests were two sided and P<0.05 was considered significant. The software package SPSS 16.0 for Windows XP was used for analysis.

## Results

### CHD1L Status in human specimens

**Figure 1:**
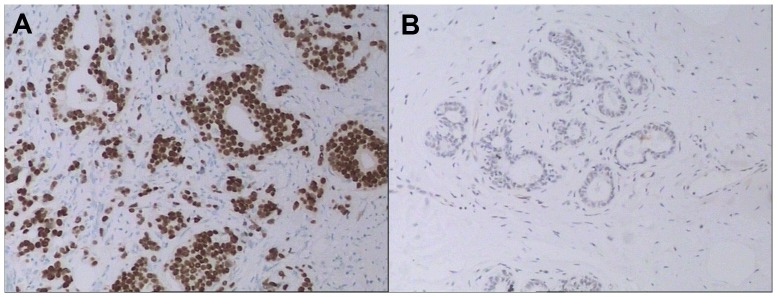
Photomicrographs for CHD1L expression. Presence of over-expression in breast cancer tissue (A) and absence of over-expression in normal breast tissue (B) (100×).

IHC staining was used to detect the expression of CHD1L protein in 179 human breast tumors and 65 matched normal breast tissue specimens. Only those tumors that contained invasive cells together with ductal structures or histologically normal breast epithelium cells were evaluated. CHD1L positive staining was seen primarily in the nuclei within tumor cells, though occasionally yellowish brown granules could be also observed in the cytoplasm. Only the intensity of the staining was taken into consideration. IRS for CHD1L expression was calculated by multiplying the PP by the corresponding SI. The median score of CHD1L protein expression in the primary breast lesions was 5, which was used as the criterion for presence of CHD1L over-expression. By using this cut-off, CHD1L was considered absence of over-expression in 92 tumors (51.4%) and presence of over-expression in 87 tumors (48.6%) (Table 1). Meanwhile, the SI of all the matched normal breast tissue were 1+ or negative, and the PP were all less than 30% (Figure 1).

**Table 1:** Association of patient and tumor characteristics with CHD1L expression.

Features	CHD1L over-expression		P Value
	Absence N(%)	Presence N(%)	
All	92(51.4)	87(48.6)	/
Age			0.011*
<55	34(37.0)	49(56.3)	
>55	58(63.0)	38(43.7)	
AJCC T Stage			0.204
1	42(45.7)	47(54.0)	
2	48(52.2)	35(40.2)	
3–4	2(2.2)	5(5.7)	
AJCC N Stage			0.928
0	52(56.5)	50(57.5)	
1	24(26.1)	23(26.4)	
2	12(13.0)	9(10.3)	
3	4(4.3)	5(5.7)	
Grade			0.004*
1	29(31.5)	11(12.6)	
2	45(48.9)	46(52.9)	
3	18(19.6)	30(34.5)	
ER			0.264
Negative	26(28.3)	32(36.8)	
Positive	66(71.7)	55(63.2)	
PR			0.456
Negative	40(43.5)	43(49.4)	
Positive	52(56.5)	44(50.6)	
HER2			0.037*
Negative	80(87.0)	64(73.6)	
Positive	12(13.0)	23(26.4)	
Ki-67	21.0±19.7	28.0±24.3	0.018*
Subtype			0.146
LA	32(34.8)	26(29.9)	
LB	34(37.0)	30(34.5)	
TN	21(22.8)	17(19.5)	
HER2+	5(5.4)	14(16.1)	

*, statistically significant.

Abbreviations: ER, estrogen receptor; PR, progesterone receptor; HER2, human epidermal growth factor receptor 2.

### CHD1L in relation to other clinical characteristics

Presence of CHD1L over-expression was more likely to be found in young breast cancer patients (P = 0.011). High grade (P = 0.004), and HER2 over-expression/amplification (P = 0.037) were significantly associated with presence of CHD1L over-expression. Patients with CHD1L over-expression had higher Ki-67 labeling index (P = 0.018). However, CHD1L expression was not related to tumor size, lymph node status, ER/PR status or molecular subtypes (Table 2).

**Table 2:** Univariate prognostic analysis of DFS and OS.

Characteristics	DFS		OS	
	Estimated mean survival(months)	P value	Estimated mean survival (months)	P value
AJCC T Stage		0.492		0.567
1	87.8		94.8	
2	82.3		91.1	
3–4	63.0		74.0	
AJCC N Stage		0.908		0.558
0	85.8		93.8	
1	82.2		92.4	
2	72.9		76.4	
3	69.0		74.8	
Grade		0.097		0.525
1	92.1		94.4	
2	80.2		92.0	
3	73.8		82.6	
ER		0.008*		0.008*
Positive	89.2		96.0	
Negative	73.2		84.1	
PR		0.014*		0.031*
Positive	90.9		96.8	
Negative	76.0		86.9	
HER2		0.145		0.148
Negative	85.9		93.4	
Positive	69.8		79.8	
Subtype		0.003*		0.018*
LA	91.8		97.9	
LB	84.9		91.5	
TN	77.0		85.7	
HER2+	53.1		68.0	
CHD1L over-expression		0.035*		0.439
Absense	88.5		90.7	
Presence	78.9		95.5	

*, statistically significant.

Abbreviations: DFS, disease free survival; OS, overall survival; ER, estrogen receptor; PR, progesterone receptor; HER2, human epidermal growth factor receptor 2; LA, luminal A; LB, luminal B; TN, triple negative.

### Association of CHD1L expression with survival of patients with breast cancer

**Figure 2:**
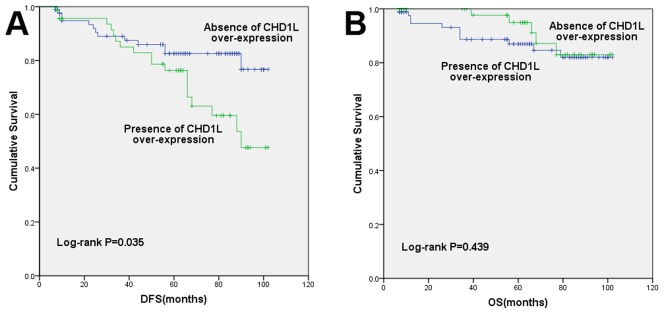
Disease-free survival (A) and overall survival (B) by CHD1L status.

After a median follow-up of 55 months (range 7–102 months), patients with presence of CHD1L over-expression had significantly poorer DFS (82.6% Vs 76.3%, P = 0.035, Fig. 2A). The estimated mean disease free interval was 88.5 months in absence of CHD1Lover-expression group while 78.9 months in presence of CHD1L over-expression group. However, no statistical significant differences could be found for OS between absence and presence of CHD1L over-expression groups (87.0%Vs 94.9%, P = 0.439, Fig. 2B). The estimated mean OS time was 90.7 months and 95.5 months, respectively.

### Univariate and multivariate analyses of prognostic variables in breast cancer patients

In univariate model, ER negativity, PR negativity, triple negative and HER2 positive subtypes, and presence of CHD1L over-expression were significantly associated with poorer DFS and all the factors, except CHD1L status, were also significantly correlated with OS (Table 2). Patients with advanced T and N stage had non-significant shorter DFS and OS, which may be due to the limited number in each T and N stratification and short follow-up period. Based on the univariate analysis results, molecular subtypes and CHD1L status were included in the multivariate Cox proportional Hazards regression model for DFS and OS. Since tumor size, axillary lymph node status and tumor grade are well-accepted as prognosis-related characteristics, these three variables were also included in the multivariate analysis. Presence of CHD1L over-expression was an independent prognostic factor for worse DFS (P = 0.042, HR = 2.169, 95%CI, 1.029–4.573) while molecular subtypes was also identified as a prognostic factor for DFS (P = 0.012, Table 3). For OS, molecular subtype was the only significant prognostic factor identified by multivariate analysis (P = 0.043, Table 3).

**Table 3:** Multivariate prognostic analysis of DFS and OS.

Characteristics	DFS		OS	
	P value	HR(95%CI)	P value	HR(95%CI)
AJCC T Stage	0.961		0.992	
1		1		1
2		0.925(0.413–2.069)		0.939(0.302–2.920)
3–4		0.800(0.140–4.564)		0.901(0.074–10.997)
AJCC N Stage	0.930		0.817	
0		1		1
1		1.058(0.430–2.607)		0.829(0.203–3.384)
2		1.150(0.309–4.272)		1.852(0.371–9.250)
3		1.708(0.351–8.300)		1.433(0.156–13.136)
Grade	0.212		0.768	
1		1		1
2		2.429(0.903–6.531)		1.618(0.441–5.930)
3		1.815(0.558–5.905)		1.321(0.281–6.212)
Subtype	0.012*		0.043*	
LA		1		1
LB	0.363	1.564(0.597–4.096)	0.316	2.153(0.480–9.652)
TN	0.033	2.809(1.086–7.264)	0.037	4.382(1.095–17.535)
HER2+	0.002	5.221(1.788–15.240)	0.009	8.645(1.715–43.587)
CHD1L over-expression	0.042*		0.479	
Absense		1		1
Presence		2.169(1.029–4.573)		0.663(0.212–2.069)

*, statistically significant.

Abbreviations: DFS, disease free survival; OS, overall survival; HR, hazard ratio; CI, confidence interval; LA, luminal A; LB, luminal B; TN, triple negative; HER2, human epidermal growth factor receptor 2.

### Expression of CHD1L in breast cancer cell lines

**Figure 3:**
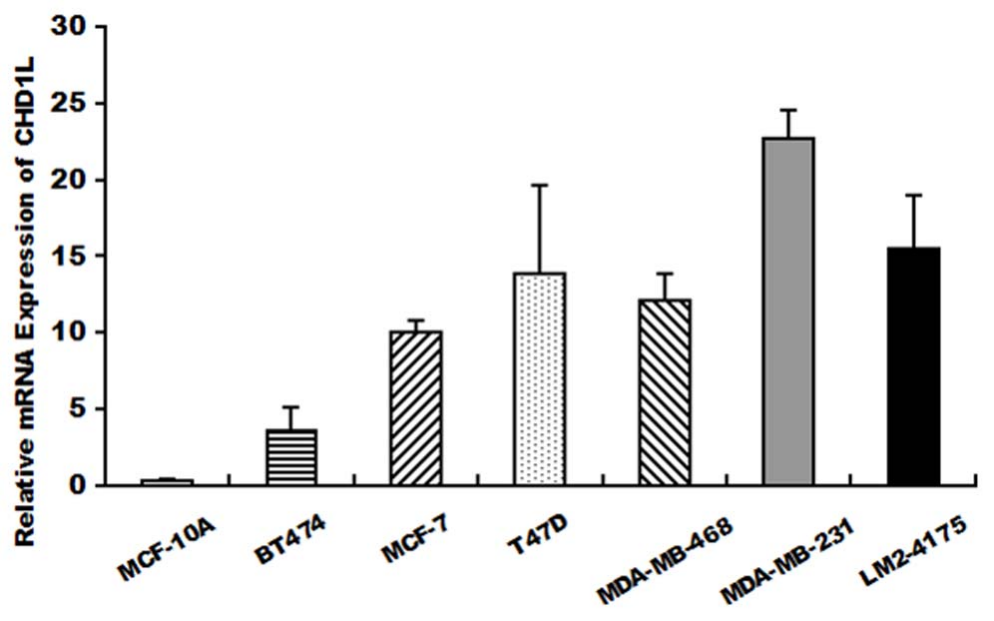
CHD1L mRNA expression levels(QRT-PCR). QRT-PCR was used to evaluate CHD1L mRNA expression levels in 6 breast cancer cell lines and the normal breast epithelial cell line MCF-10A.

Expression of the mRNA level of CHD1L was analyzed in one normal breast epithelial cell line and six breast cancer cell lines by using QRT-PCR. The results showed that the levels of CHD1L mRNA were relatively higher in breast cancer cell lines when compared to MCF-10A, especially in MDA-MB-231 and LM2-4175. (Fig. 3)

## Discussion

Previous researches from CGH analysis indicate that amplification of 1q21 is the most frequent genetic alteration in HCC [21]–[24]. A novel oncogene, CHD1L (also named ALC1), has been isolated at 1q21 and identified that over-expression of CHD1L could promote G1/S phase transition and inhibit apoptosis [5]. However, to date, the expression of CHD1L in breast cancer has not been investigated, thus the clinico-pathological and prognostic value of which remains unknown.

To our knowledge, this is the first study to explore the relationship between CHD1L expression and breast cancer characteristics, subtypes and patient survival in a cohort of primary breast cancers. In our study, presence of CHD1L over-expression was found in 48.6% of the breast cancer patients. We also demonstrated that presence of CHD1L over-expression was associated with younger age, higher tumor grade, HER2 over-expression/amplification, and higher Ki-67 labeling index, which are all prognostic factors of unfavorable breast cancer biology. Meanwhile, CHD1L expression was not related to tumor stage in our study, which is in line with previous findings in HCC and ovarian carcinoma [12], [20]. In these two studies, similar positive rates of CHD1L over-expression were reported (50.5% for HCC and 51% for ovarian carcinoma). Likewise, CHD1L expression was found associated with tumor histopathology and grade, but not tumor stage in HCC and ovarian carcinoma [20], [25]. Combined, previous research work supported the hypothesis that CHD1L expression was more likely to be correlated with tumor biology rather than tumor burden.

It was previously reported that presence of CHD1L over-expression was significantly associated with poorer DFS and OS in both HCC and ovarian carcinoma [12], [20]. In our study, we reported that presence of CHD1L over-expression was significantly correlated with worse DFS (82.6% Vs 76.3%, P = 0.035) by Kaplan-Meier analysis. In multivariate analysis, both molecular subtypes (P = 0.012) and presence of CHD1L over-expression (P = 0.044, HR = 2.174, 95%CI, 1.020–4.634) were independent predictors of DFS, but not OS, which may be due to limited follow-up or various treatments after first relapse of the disease. This finding may indicate that CHD1L plays an important role in progression and metastasis of breast cancer. Recent gene expression studies have confirmed that breast cancer is no longer a single disease with variable morphology, but with high molecular heterogeneity [26]. Gene profiling has already identified at least five intrinsic subtypes of breast cancer, with different tumor biology, treatment response, and prognosis [13], [14]. However, heterogeneity still exists in each intrinsic subtype and current surrogate definitions of intrinsic subtypes based on IHC results has been questioned on its accuracy in mimicking gene expression-based assays [27]. Thus, it is important to add novel biomarkers to ‘subgroup’ them furthermore. According to our results, immunohistochemical staining status of CHD1L expression might be useful as an additional biomarker to identify those breast cancer patients with increased risk of relapse.

Also, we evaluated the expression of CHD1L in breast cancer cell lines. The expression level of CHD1L is relatively higher in breast cancer cell lines than in MCF-10A, the normal breast epithelial cell line which suggested that CHD1L might have an oncogenic ability. Meanwhile, the highest CHD1L levels were found in MDA-MB-231, a post-epithelial-mesenchymal transition(post-EMT) subtype cell line [28], [29] with stem cell-like features [30] and LM2-4175, a lung metastatic subpopulation from MDA-MB-231 with mesenchymal features [19], [31]. Since it has been reported that patients with mesenchymal-like breast cancer had a worse outcome [30], [32], [33], our results in vitro somehow confirmed that CHD1L expression was related to an aggressive breast cancer biology and might also indicate the probability of CHD1L playing a role in EMT of breast cancer.

However, the mechanism of CHD1L-related breast cancer development has not yet been revealed. Ma and his colleagues first reported that over-expression of CHD1L could promote G1/S phase transition and inhibit apoptosis [5]. Further studies demonstrated the oncogenic function of CHD1L in HCC development and progression via several signal pathways in both vitro and in vivo experiments [10], [11], [34]. It is also reported that CHD1L is activated by poly (ADP-ribose) (PAR) polymerase-1 (PARP-1), a DNA nick sensor enzyme that is activated by DNA breaks, and rapidly recruited to DNA damage sites specifically induced by PAR [35]. In addition, CHD1L activity can be blocked by PARP-1 inhibitors, which have already been explored for use in cancer treatment [36]. These findings offer a novel concept that PARP-1 inhibitor might be an efficient therapeutic agent to treat CHD1L-overexpressed breast cancer. Clearly, further studies are warranted to demonstrate the oncogenic function of CHD1L in breast cancer and the mechanisms how CHD1L promotes the development and metastasis of breast cancer.

## Conclusions

In summary, the current study highlights for the first time that the presence of CHD1L over-expression in breast cancer patients, as measured by immunohistochemical analyses, is more likely to be associated with aggressive tumor biology rather than large tumor burden in breast cancer. Presence of CHD1L over-expression might be a novel prognostic biomarker for patients with breast cancer. In vitro study in cell lines somehow confirmed these results. Further basic and translational studies are warranted to demonstrate the mechanisms how CHD1L promotes tumor development in breast cancer.
